# Correction: Breadth and function of antibody response to acute SARS-CoV-2 infection in humans

**DOI:** 10.1371/journal.ppat.1010148

**Published:** 2021-12-10

**Authors:** Kuan-Ying A. Huang, Tiong Kit Tan, Ting-Hua Chen, Chung-Guei Huang, Ruth Harvey, Saira Hussain, Cheng-Pin Chen, Adam Harding, Javier Gilbert-Jaramillo, Xu Liu, Michael Knight, Lisa Schimanski, Shin-Ru Shih, Yi-Chun Lin, Chien-Yu Cheng, Shu-Hsing Cheng, Yhu-Chering Huang, Tzou-Yien Lin, Jia-Tsrong Jan, Che Ma, William James, Rodney S. Daniels, John W. McCauley, Pramila Rijal, Alain R. Townsend

There is an error in the caption for S2 Fig. The cells in this experiment were transduced to express the unmutated full length wild-type spike protein and did not contain the stabilizing mutations as stated in the figure legend. Please see the full S2 Fig legend below.

There is also an error in the last sentence of the second paragraph of the Results subsection “Genetic and phenotypic characteristics of anti-spike glycoprotein antibodies”. The correct sentence is: All anti S2 MAbs bound to the MDCK-SIAT1 cells transduced to express the full-length spike antigen.

## Supporting information

S2 Fig The binding activity of anti-SARS-CoV-2 S2 antibodies with spike-expressed MDCK cells in flow cytometry. We produced MDCK-Spike by stably transducing parental MDCK-SIAT1 cells with cDNA expressing full-length unmutated SARS-CoV-2 spike glycoprotein. MDCK-H3 cells were stained in the control experiment. Anti-influenza H3 MAb BS-1A was included as an antibody control. Each experiment was repeated twice (n = 2). The binding percentage was presented as mean ± standard error of the mean.
(TIF)
