# Supplementary material for: Breadth and function of antibody response to acute SARS-CoV-2 infection in humans
Source: PLoS Pathog. 2021 Feb 26;17(2):e1009352. doi: 10.1371/journal.ppat.1009352 (PMC8130932; doi:10.1371/journal.ppat.1009352)
Supplement: S1 Table — (DOC) [file ppat.1009352.s001.doc]

| **S1 Table. Clinical characteristics of COVID-19 patients and sampling dates in the study.** | | | | | | | | | | | |
| --- | --- | --- | --- | --- | --- | --- | --- | --- | --- | --- | --- |
|  | **Age (yrs)** | | **Gender** | **Onset symptoms** | **Pneumonia** | **Oxygen  use** | **Ventilator  use** | **Fever subside** | **Sequela** | **Plasmablast sampling dates** | **Serum sampling dates** |
| **Case A** | 43 | M | | Fever, headache | Day 7  after onset | Yes | none | Day 9 after onset | none | Day 14  after onset | Day 22  after onset |
|  |  |  | |  |  |  |  |  |  |  |  |
| **Case B** | 55 | F | | Cough, fever | Day 11  after onset | Yes | none | Day 20 after onset | none | Days 14, 18 and 22  after onset | Days 14, 18 and 26  after onset |
|  |  |  | |  |  |  |  |  |  |  |  |
| **Case C** | 52 | M | | Fever | none | none | none | Day 1 after onset | none | Days 2, 6 and 14  after onset | Days 6, 10, 14 and 18  after onset |
