# Supplementary material for: Breadth and function of antibody response to acute SARS-CoV-2 infection in humans
Source: PLoS Pathog. 2021 Feb 26;17(2):e1009352. doi: 10.1371/journal.ppat.1009352 (PMC8130932; doi:10.1371/journal.ppat.1009352)
Supplement: S2 Table — (DOC) [file ppat.1009352.s002.doc]

| **S2 Table. Anti-SARS-CoV-2 spike monoclonal antibody heavy and light chain variable domain gene usage.** | | | | | | | | | | | | | |
| --- | --- | --- | --- | --- | --- | --- | --- | --- | --- | --- | --- | --- | --- |
| MAb | H-L | Vh | Jh | Dh | rf | V_h_ junction sequence | nt Mut | aa Sub | Vl | Jl | V_l_ Junction Sequence | nt Mut | aa Sub |
| FM 7B | H-𝝺 | 1-3*04 F | 5*02 F | 2-2*01 F | 2 | CARDPTYCSSTSCYPFSWFDPW | 3 | 1 | 3-21*02 F | 3*02 F | CQVWDSTGDHSWVF | 2 | 2 |
| FD 8B | H-K | 1-24*01 F | 6*02 F | 2-2*01 F | 2 | CATAAAINCSSTSCYYYYYYYGMDVW | 0 | 0 | 2-24*01 F | 2*01 F | CTQATQFPYTF | 2 | 2 |
| EW 9B | H-K | 1-46*01 or 03 F | 6*02 F | 2-2*01 F | 3 | CAREDGVVPAANLMISLEDYYYYGMDVW | 2 | 2 | 3-11*01 F | 4*01 F | CQQRSNWPLTF | 0 | 0 |
| FN 12A | H-𝝺 | 1-69*04 or 09 F | 6*02 F | 2-2*01 F | 2 | CARSGCSSTSCPSNLYYYYYGMDVW | 1 | 0 | 1-51*01 F | 3*02 F | CGTWDSSLSALVF | 1 | 0 |
| FG 12C | H-𝝺 | 3-9*01 F | 4*02 F | 4-17*01 F | 2 | CAKDMRVHDYGDYYFDYW | 0 | 0 | 3-21*01 F | 2*01 or 3*01 F | CQVWDSSSDHPVF | 1 | 1 |
| FI 1C | H-𝝺 | 3-11*04 F | 3*02 F | 6-13*01 F | 1 | CARRSNRFLIAFDIW | 3 | 2 | 2-14*01 F | 2*01 or 3*01 F | CSSYTSSSTLVVF | 1 | 1 |
| FI 4A | H-𝝺 | 3-21*01 F | 4*02 F | 2-21*02 F | 2 | CATYLFGDSHTYW | 9 | 7 | 6-57*02 F | 3*02 F | CQSYDSSNLHWVF | 0 | 0 |
| FD 1E | H-𝝺 | 3-21*01 F | 6*02 F | 6-13*01 F | 2 | CASLAAAGPETYYYYGMDVW | 1 | 0 | 3-1*01 F | 2*01 or 3*01 F | CQAWDSSVVF | 0 | 0 |
| FD 11E | H-𝝺 | 3-21*01 F | 6*02 F | 6-13*01 F | 2 | CASLAAAGPETYYYYGMDVW | 0 | 0 | 3-1*01 F | 2*01 or 3*01 F | CQAWDSSVVF | 0 | 0 |
| FN 2C | H-𝝺 | 3-30*03 or 18 or 3-30-5*01 F | 3*01 or 02 F | 3-10*01 F | 1 | CAKRREIFWLGEPPLSDAFDFW | 22 | 14 | 1-40*01 F | 3*02 F | CQSYDSSLSGSVF | 8 | 4 |
| EY 6A | H-K | 3-30*03 or 18 or 3-30-5*01 F | 4*02 F | 2-21*01 F | 1 | CAKDGGKLWVYYFDYW | 6 | 5 | 1-39*01 F or 1D-39*01 F | 4*01 F | CQQSYSTLALTF | 0 | 0 |
| FD 11D | H-K | 3-30*03 or 18 or 3-30-5*01 F | 4*02 F | 6-19*01 F | 1 | CAKEGAGSGWYRHHKPGYYFDYW | 1 | 1 | 3-20*01 F | 1*01 F | CQQYGSSPLTF | 0 | 0 |
| EW 9C | H-K | 3-30*03 or 18 or 3-30-5*01 F | 5*01 or 02 F | 3-10*01 F | 1 | CARATSIFWFGEGRNWFDPW | 33 | 15 | 3-11*01 F | 4*01 F | CQQRSNWPLTF | 25 | 13 |
| FD 1D | H-K | 3-30*04 or 3-30-3*03 F | 5*02 F | 3-10*01 F | 2 | CARAGSGSYLNWFDPW | 1 | 0 | 3-11*01 F | 5*01 F | CQQRSNWPITF | 0 | 0 |
| FG 7A | H-K | 3-30-3*01 F | 4*02 F | 1-26*01 F | 3 | CARSHSGSYRASLDYW | 2 | 2 | 3-20*01 F | 2*01 F | CQQYGSSPLYTF | 0 | 0 |
| FM 1A | H-𝝺 | 3-33*01 or 06 F | 4*02 F | 1-26*01 F | 1 | CAREGAVGATRGFDYW | 1 | 0 | 3-21*02 F | 2*01 or 3*01 F | CQVWDSSSDQGVF | 2 | 1 |
| FD 11A | H-𝝺 | 3-33*01 or 06 F | 6*02 F | 3-9*01 F | 2 | CAKGPDILTGYYNYYYYGMDVW | 2 | 2 | 1-40*01 F | 2*01 or 3*01 F | CQSYDSSLSGFYVVF | 0 | 0 |
| FN 8C | H-𝝺 | 3-33*05 F | 6*02 F | 3-9*01 F | 2 | CARERTYYDILTGYRHYYGMDVW | 1 | 1 | 3-21*02 F | 3*02 F | CQVWDSSSDHWVF | 1 | 1 |
| FD 5E | H-K | 3-43D*03 F | 6*02 F | 3-3*01 F | 1 | CAKDSVRFRYYYGMDVW | 0 | 0 | 3-11*01 F | 3*01 F | CQQRSNWPLTF | 0 | 0 |
| FD 5D | H-K | 3-48*04 F | 6*02 F | 6-13*01 F | 2 | CASPGGITAAGTSVLFGYYGMDVW | 4 | 2 | 2-28*01 or 2D-28*01 F | 1*01 F | CMQALQTPITWTF | 0 | 0 |
| FI 3A | H-K | 3-53*01 F | 3*02 F | 6-6*01 F | 3 | CARDHVRPGMNIW | 2 | 2 | 1-33*01 or 1D-33*01 F | 4*01 F | CQQYDNLPVTF | 1 | 0 |
| FD 10A | H-K | 3-74*01 F | 3*02 F | - |  | CANMAFDIW | 0 | 0 | 4-1*01 F | 5*01 F | CQQYYSTPITF | 0 | 0 |
| FJ 4E | H-K | 4-31*06 F | 5*02 F | 3-10*01 F | 2 | CARDEYDSSDSGIQGHWFDPW | 20 | 15 | 1-39*01 F or 1D-39*01 F | 1*01 F | CQQSYSTPWTF | 15 | 10 |
| FJ 1C | H-𝝺 | 4-38-2*02 F | 4*02 F | 3-10*01 F | 1 | CARDKALLWFGELFTNLFDYW | 0 | 0 | 2-14*01 F | 2*01 or 3*01 F | CSSYTSSSTLVF | 1 | 1 |
| EW 8B | H-K | 4-39*01 F | 3*02 F | 3-16*01 F | 2 | CARQEVWGGFDIW | 20 | 12 | 3-20*01 F | 1*01 F | CQQYGSSPTF | 4 | 4 |
| FD 11C | H-𝝺 | 4-39*07 F | 6*02 F | 3-10*01 F | 2 | CAREYYYGSETKKYYYYYGMDVW | 2 | 1 | 3-1*01 F | 2*01 or 3*01 F | CQAWDSSTVF | 0 | 0 |
| FD 7C | H-K | 4-59*01 F | 5*02 F | 3-10*02 F | 3 | CARDYRFGELFGRFAWFDPW | 1 | 1 | 3-15*01 F | 1*01 F | CQQYNNWPRAF | 2 | 1 |
| FD 7D | H-𝝺 | 5-10-1*03 F | 3*02 F | 2-2*01 F | 2 | CARHSDCSSTSCYFVDAFDIW | 1 | 1 | 3-25*03 F | 2*01 or 3*01 F | CQSADSSGTYVVF | 1 | 0 |
| FJ 10B | H-K | 5-10-1*03 F | 6*02 F | 3-16*01 F | 1 | CARLDPRYGPDYYGMDVW | 2 | 1 | 1-39*01 F or 1D-39*01 F | 4*01 F | CQQSYSTPLTF | 3 | 2 |
| FB 9D | H-𝝺 | 5-51*01 F | 6*02 F | 3-10*01 F | 3 | CARHWASMVRGVIRASHYYGMDVW | 27 | 11 | 2-8*01 F | 3*02 F | CSSYAFGGSDTRVF | 20 | 10 |
| FB 1E | H-𝝺 | 5-51*01 F | 6*02 F | 3-10*01 F | 3 | CARHWASMVRGVIRASHYYGMDVW | 22 | 11 | 2-8*01 F | 3*02 F | CSSYAFGGSDIRVF | 16 | 8 |
| EZ 7A | H-𝝺 | 5-51*01 F | 6*02 F | 5-12*01 F | 3 | CARGWVYRGFPYYGMDVW | 2 | 1 | 2-11*01 F | 3*02 F | CCSYAGSYTLVF | 0 | 0 |

Abbreviations: H, heavy; K, kappa; 𝝺, lambda; Vh, variable gene segment of the heavy chain variable domain; Dh, diversity gene segment of the heavy chain variable domain; Jh, joining gene segment of the heavy chain variable domain; Mut, number of nucleotide mutations; Sub, number of amino acid substitutions; Vl, variable gene segment of the light chain variable domain; Jl, joining gene segment of the light chain variable domain.
