# Supplementary material for: Breadth and function of antibody response to acute SARS-CoV-2 infection in humans
Source: PLoS Pathog. 2021 Feb 26;17(2):e1009352. doi: 10.1371/journal.ppat.1009352 (PMC8130932; doi:10.1371/journal.ppat.1009352)
Supplement: S3 Table — (DOC) [file ppat.1009352.s003.doc]

| **S3 Table. Anti-SARS-CoV-2 nucleocapsid monoclonal antibody heavy and light chain variable domain gene usage.** | | | | | | | | | | | | | |
| --- | --- | --- | --- | --- | --- | --- | --- | --- | --- | --- | --- | --- | --- |
| MAb | H-L | Vh | Jh | Dh | rf | V_h_ junction sequence | nt Mut | aa Sub | Vl | Jl | V_l_ Junction Sequence | nt Mut | aa Sub |
| EZ 11A | H-𝝺 | 3-7*01 F | 4*02 F | 1-26*01 F | 3 | CARDDYSGSYYWEFDYW | 0 | 0 | 4-69*01 F | 3*02 F | CQTWGTGIWVF | 0 | 0 |
| FD 6D | H-K | 4-38-2*02 F | 3*01 or 02 F | 3-22*01 F | 2 | CARDRLLAVHYDSRGYLVDYW | 22 | 10 | 4-1*01 F | 1*01 F | CQQYYDIPRTF | 10 | 6 |
| EZ 7B | H-K | 4-59*13 F | 3*02 F | 1-26*01 F | 3 | CARRVFGPVLPSKLGGSYWGGGAFDIW | 1 | 1 | 4-1*01 F | 3*01 F | CQQYYSTPLTF | 0 | 0 |
| EZ 4C-1 | H-K | 4-61*01 or 03 F | 4*02 F | 3-3*01 F | 1 | CARAPSAPFGGLFDWILPKGINNW | 24 | 15 | 1-5*03 F | 2*03 F | CQQYNGYSYSF | 17 | 8 |
| EY 2A | H-K | 5-51*01 F | 4*02 F | 6-13*01 F | 1 | CVRQERGSNTWYAGNSW | 45 | 23 | 2-28*01 or 2D-28*01 F | 2*02 F | CMQALQTPGTF | 14 | 7 |
| EY 3B | H-K | 5-51*01 F | 4*02 F | 6-13*01 F | 1 | CVRQERGSNTWYAGNSW | 42 | 22 | 2-28*01 or 2D-28*01 F | 2*02 F | CMQALQTPGTF | 13 | 7 |
| EZ 4A | H-K | 5-51*01 F | 4*02 F | 6-13*01 F | 2 | CARSPIAADLFDYW | 0 | 0 | 1-33*01 or 1D-33*01 F | 3*01 F | CQQYDNLLFTF | 0 | 0 |
| FB 9B | H-𝝺 | 7-4-1*02 F | 4*02 F | 3-10*01 F | 1 | CARGSGTWFGELLDYW | 11 | 8 | 2-8*01 F | 2*01 or 3*01 F | CASYVGSSKLIF | 15 | 9 |

Abbreviations: H, heavy; K, kappa; 𝝺, lambda; Vh, variable gene segment of the heavy chain variable domain; Dh, diversity gene segment of the heavy chain variable domain; Jh, joining gene segment of the heavy chain variable domain; Mut, number of nucleotide mutations; Sub, number of amino acid substitutions; Vl, variable gene segment of the light chain variable domain; Jl, joining gene segment of the light chain variable domain.
