# Supplementary figures and images for: Breadth and function of antibody response to acute SARS-CoV-2 infection in humans
Source: PLoS Pathog. 2021 Feb 26;17(2):e1009352. doi: 10.1371/journal.ppat.1009352 (PMC8130932; doi:10.1371/journal.ppat.1009352)

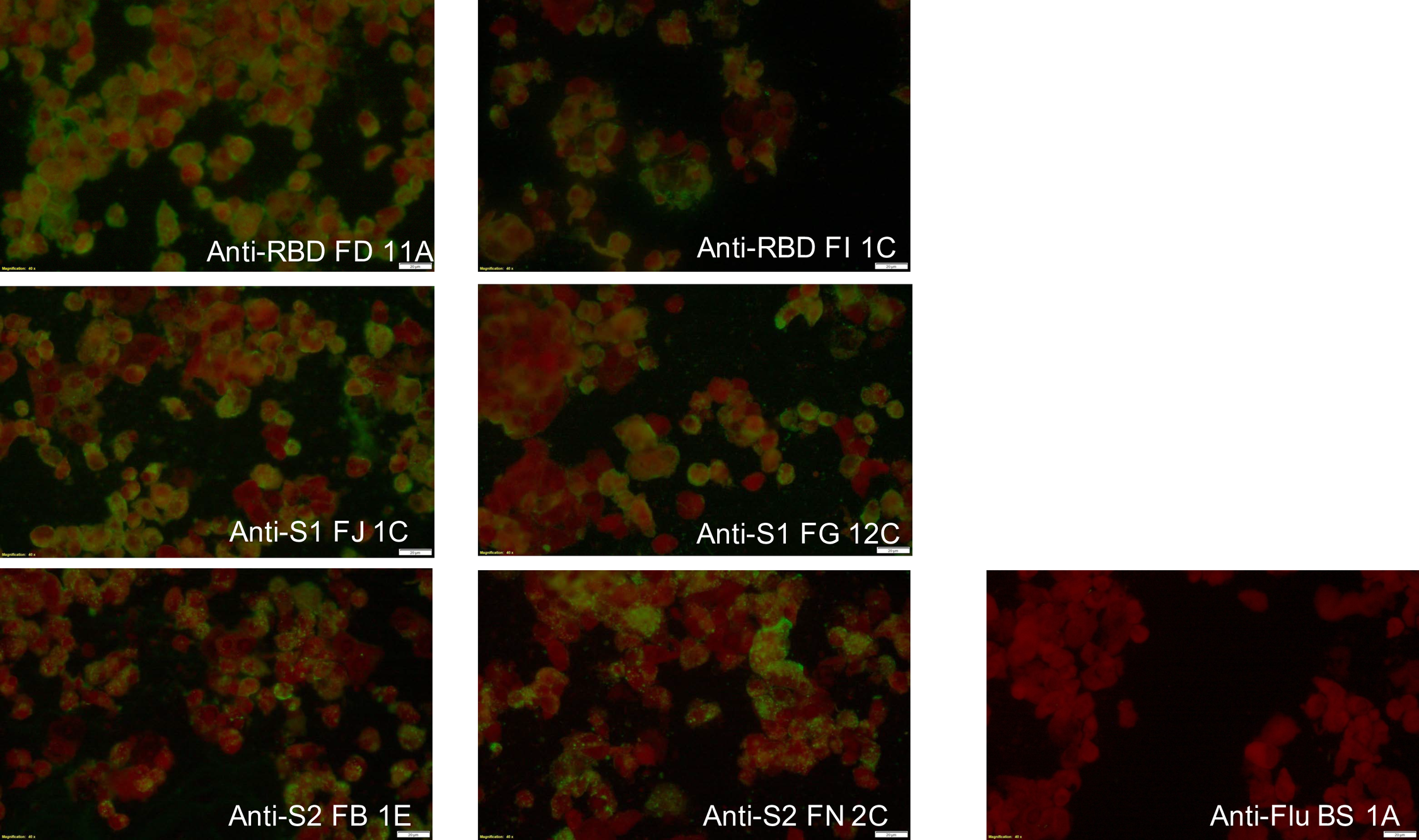

Supplement: S1 Fig — Representative immunofluorescence staining of anti-RBD, anti-S1 and anti-S2 MAbs are shown as apple-green fluorescence a background of red fluorescing material stained by Evans Blue counterstain. Anti-influenza H3 MAb BS 1A was included as a control. Images were acquired with original magnification 40x, scale bar 20 μm. (TIF) [file ppat.1009352.s004.tif]

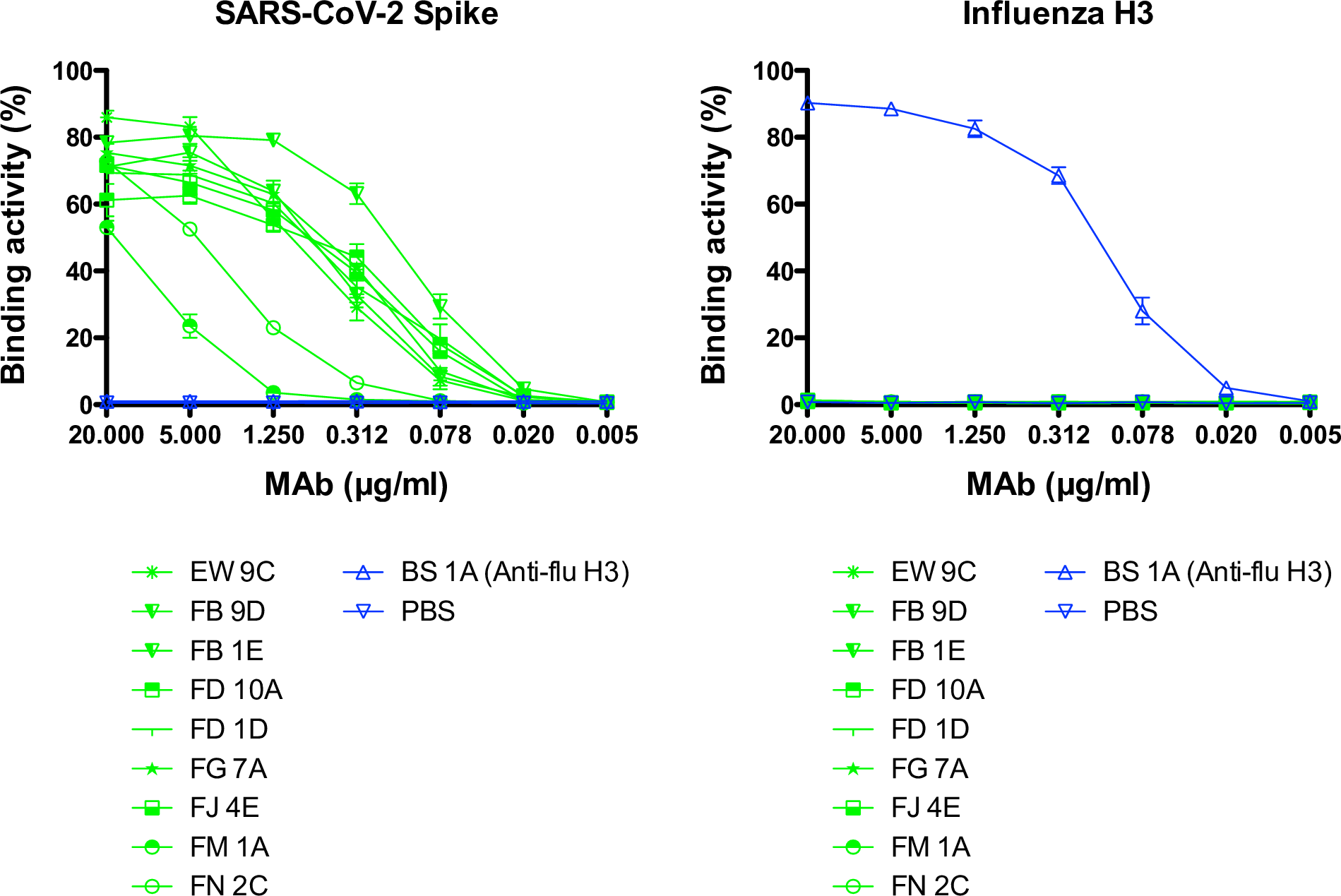

Supplement: S2 Fig — We produced MDCK-Spike by stably transducing parental MDCK-SIAT1 cells with cDNA expressing full-length SARS-CoV-2 spike glycoprotein. This cell membrane-bound full-length spike carries trimer-stabilizing proline mutations (986KV987 to 986PP987) and substitutions at the S1-S2 furin cleavage site (682RRAR685 to 682GSAG685), which indicates that the spike would display the prefusion conformation. MDCK-H3 cells were stained in the control experiment. Anti-influenza H3 MAb BS-1A was included as an antibody control. Each experiment was repeated twice (n = 2). The binding percentage was presented as mean ± standard error of the mean. (TIF) [file ppat.1009352.s005.tif]

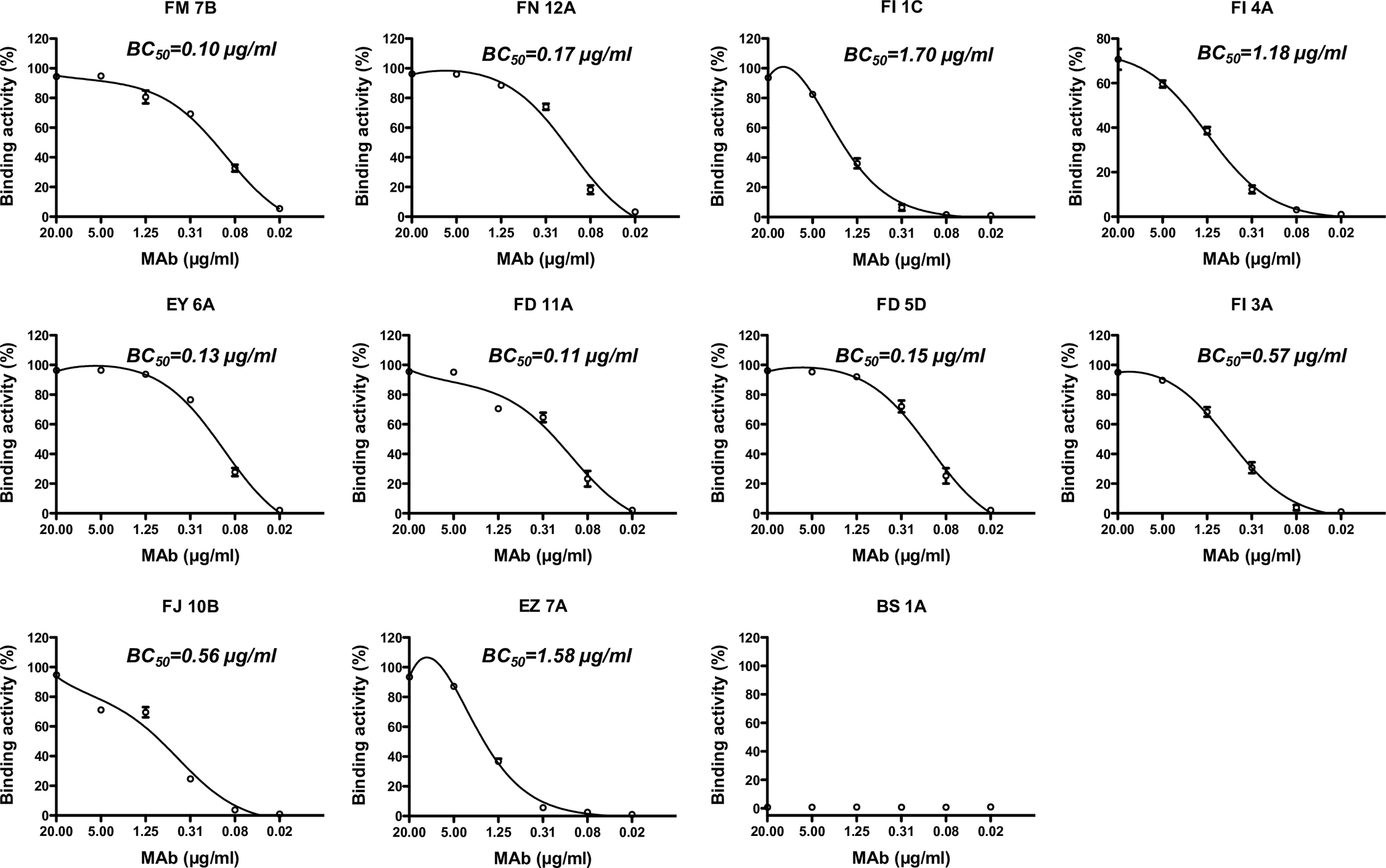

Supplement: S3 Fig — Anti-influenza H3 MAb BS-1A was included as a control. Binding percentages are presented as mean ± standard error of the mean. Each experiment was repeated twice (n = 2). The 50% binding concentration (BC50) was measured with a curve fit using non-linear regression. (TIF) [file ppat.1009352.s006.tif]

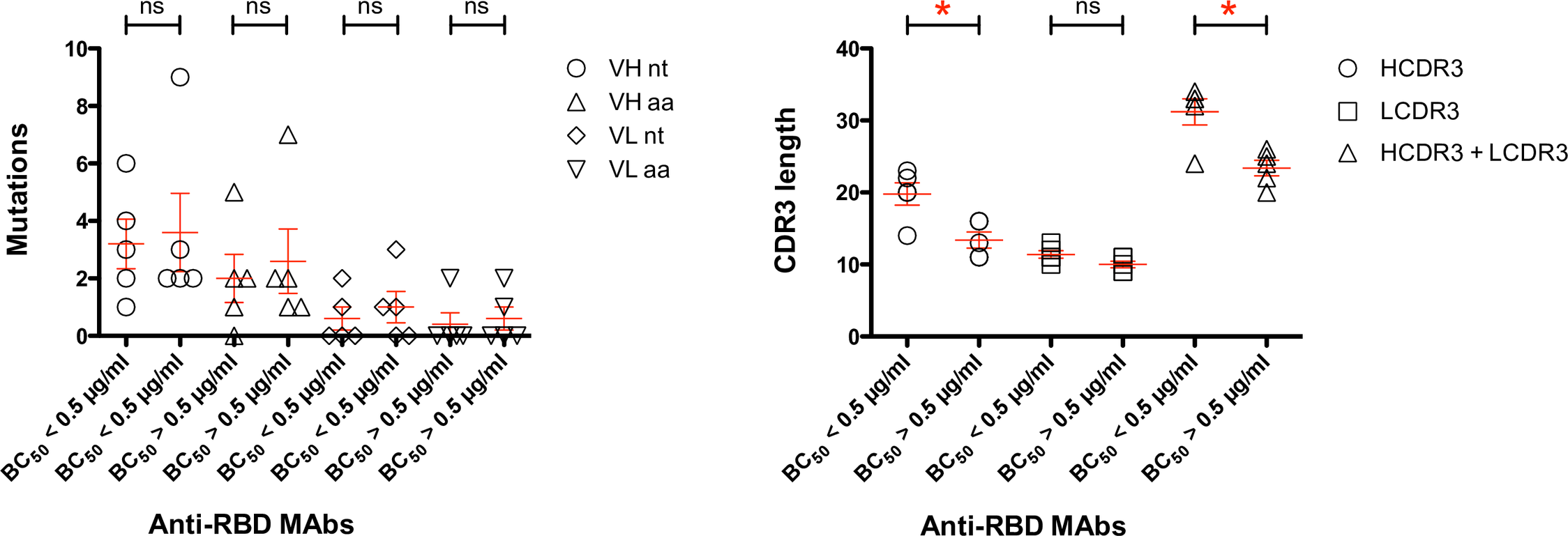

Supplement: S4 Fig — The CDR3 length (number of amino acids) and MAb gene mutation numbers are presented as mean ± standard error of the mean (< 0.5 μg/ml, n = 5 versus > 0.5 μg/ml, n = 5). The two-tailed Mann-Whitney test was performed to compare the CDR3 length and mutation numbers between two groups. * P < 0.05; ns, non-significant; BC50, 50% binding concentration. (TIF) [file ppat.1009352.s007.tif]

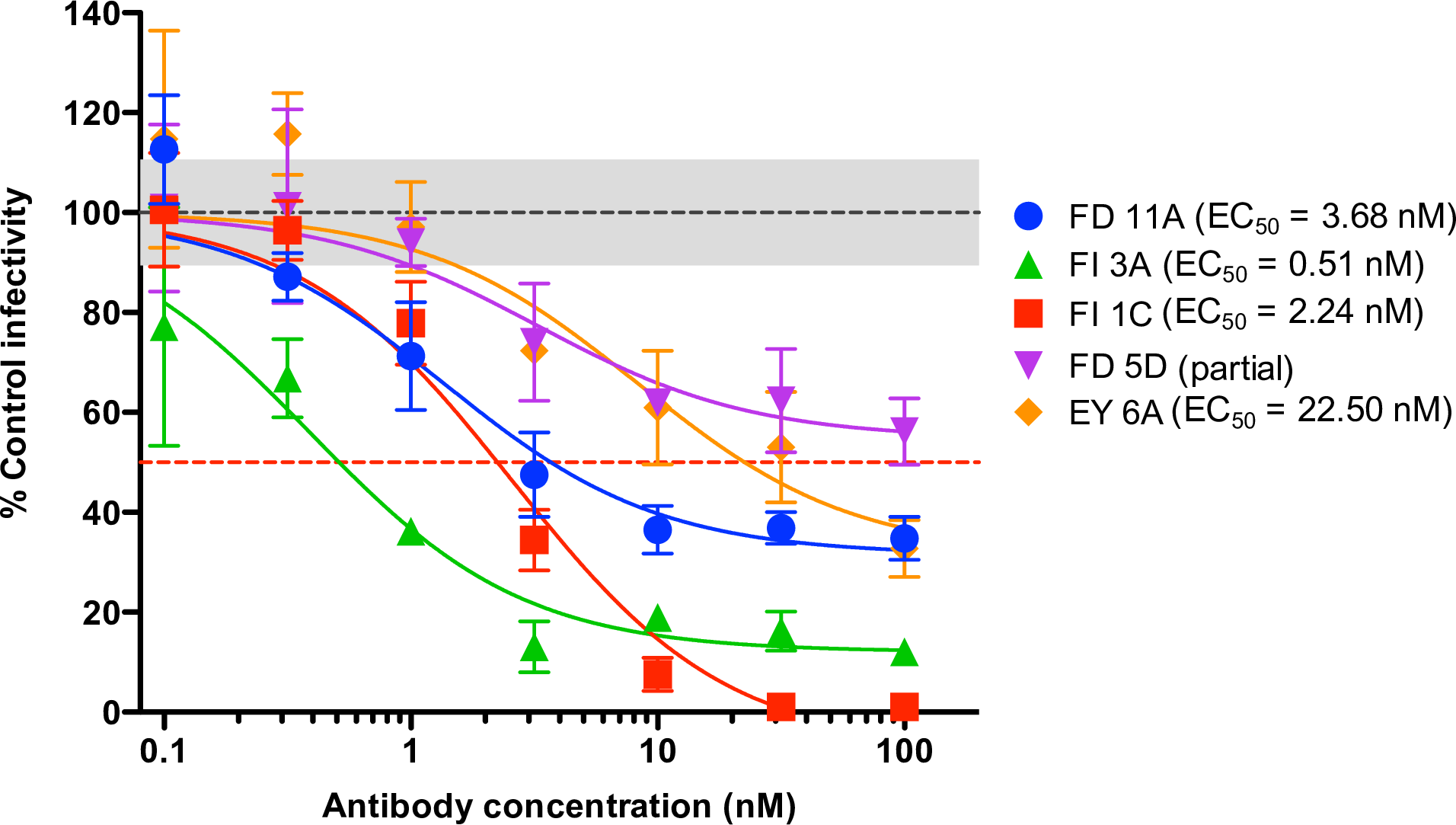

Supplement: S5 Fig — Neutralisation assays were performed on the indicated antibodies according to the fluorescent focus-forming units microneutralisation method (see methods). Data were normalized to control (no antibody) values of foci, and the grey region comprises ± 1 standard deviation the mean control values. Individual points are displayed ± 1 standard deviation of technical, and curves are shown only where the data for a particular antibody fitted the standard dose-response (Hill) equation (n = 3). Partial: MAb neutralises at least ~40% viruses at 100 nM (highest concentration tested). EC50, 50% effective concentration. (TIF) [file ppat.1009352.s008.tif]

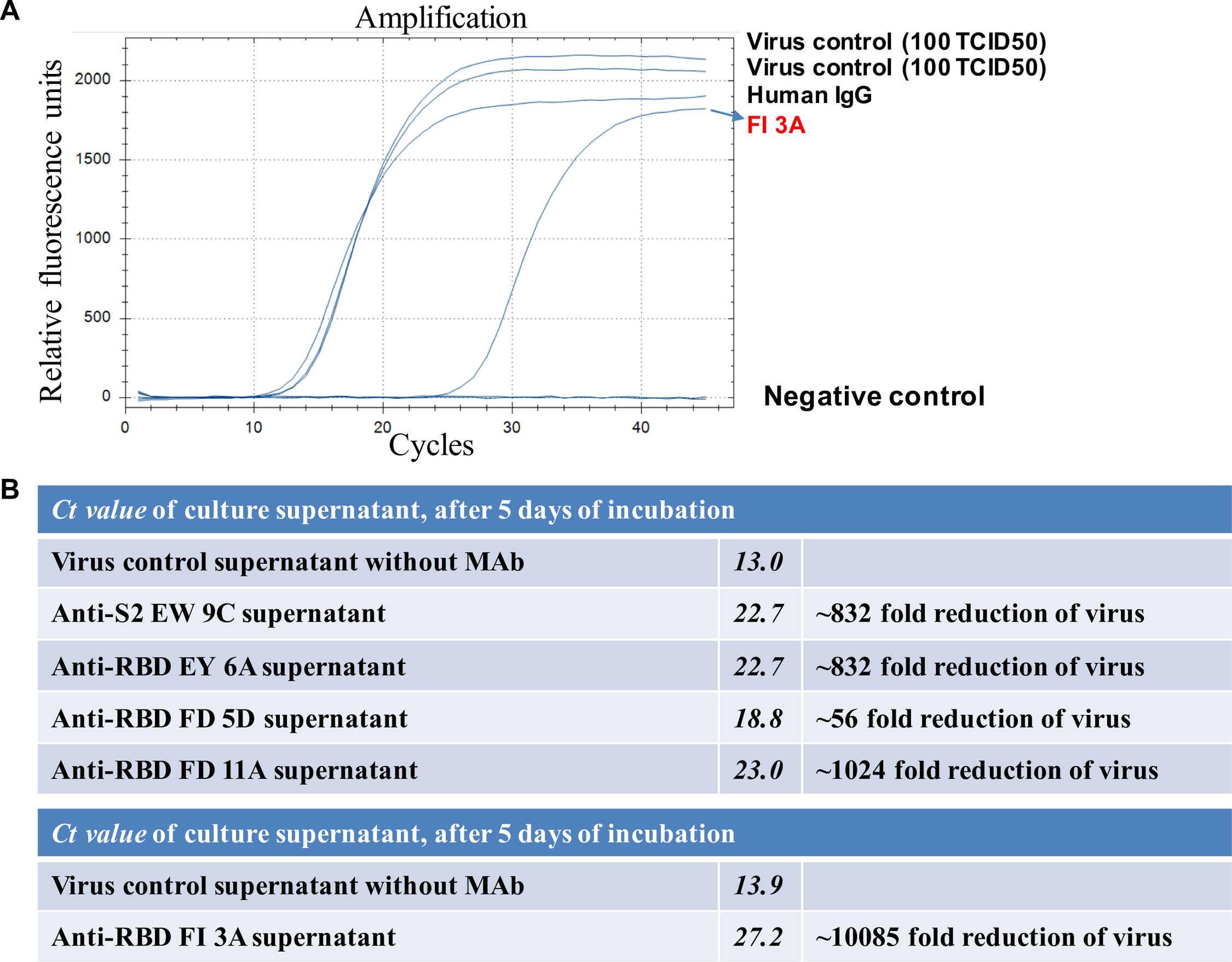

Supplement: S6 Fig — (A) An illustrative example of measuring Ct value of virus signal in the tissue-culture supernatant of SARS-CoV-2 infected Vero E6 cells using an E gene-based real-time reverse-transcription PCR assay. The right shift of the amplification plot reflects the increase in Ct value and the decrease of viral load. (B) Neutralisation data for MAbs EW 9C, EY 6A, FD 5D, FD 11A and FI 3A. Increases in Ct value indicate decreases in virus loads. Each unit increase indicates a 2x reduction resulting from the presence of MAb. A 10x increase in Ct = 1,024-fold reduction of virus load. (TIF) [file ppat.1009352.s009.tif]

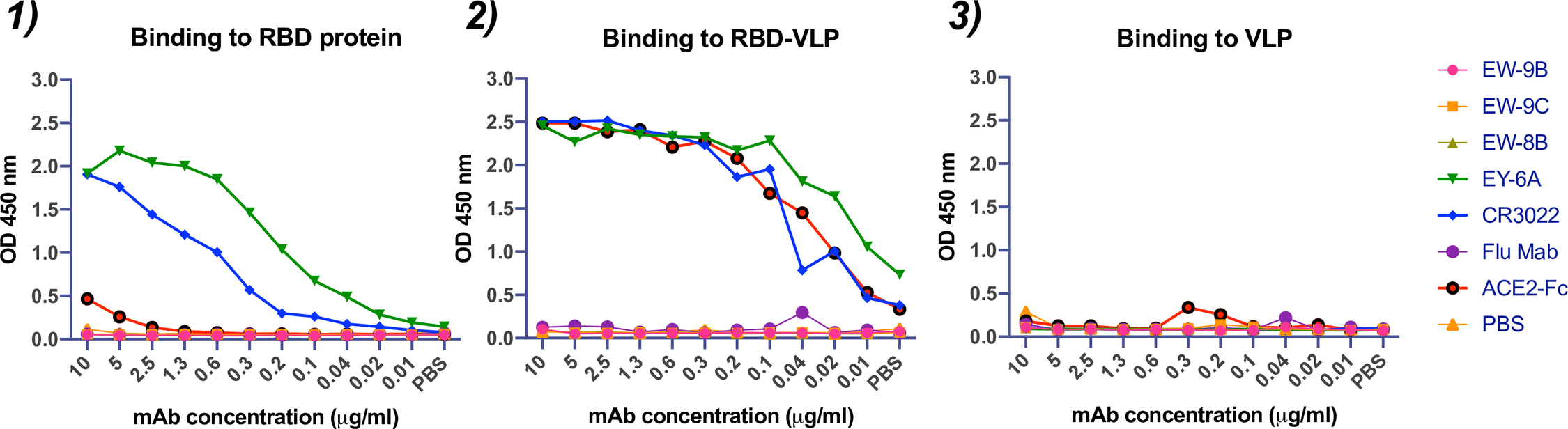

Supplement: S7 Fig — RBD bound directly to plate (Panel 1) fails to bind ACE2-Fc, but RBD-VLP bound to plate (Panel 2) exposes the ACE2 binding site on RBD, and the epitopes bound by the MAbs CR3022 and EY 6A. VLP only (Panel 3) was included as a control in the assay. Other MAbs EW 9B, EW 9C, EW 8B bind elsewhere on the spike glycoprotein (therefore are negative in this assay), anti-influenza H7 haemagglutinin MAb is a negative control. Each experiment was repeated twice. (TIF) [file ppat.1009352.s010.tif]

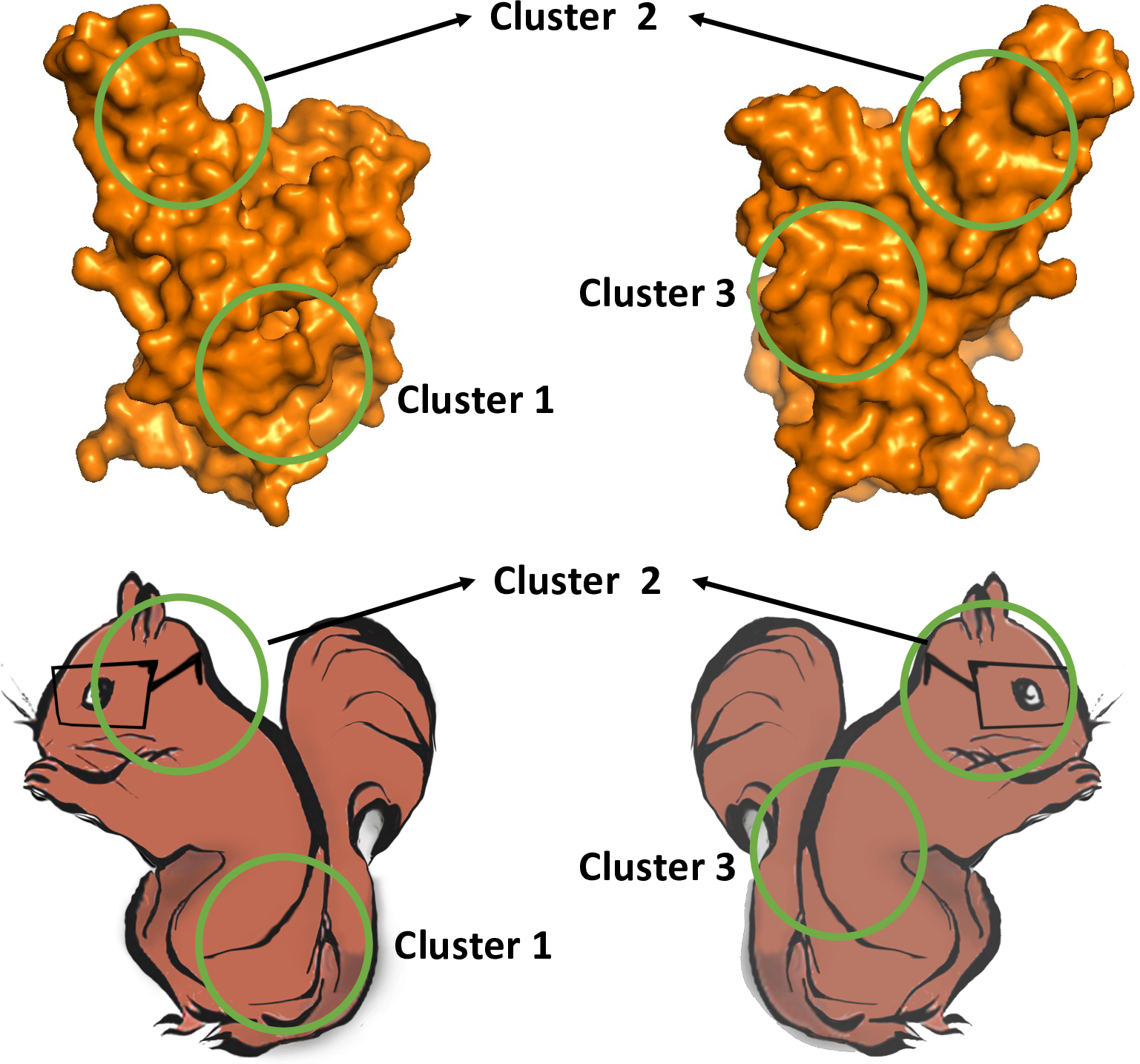

Supplement: S8 Fig — Cluster 2 antibodies is composed of antibodies in the Barnes Classes 1 and 2 antibodies that bind to the left and ride side of the head/shoulder of the “squirrel” RBD [20]. Cluster 1 antibodies that correspond to Barnes Class 4 antibodies bind to the left hip of the “squirrel” RBD. Cluster 3 antibodies that correspond to Barnes Class 3 antibodies bind to the right hip of the “squirrel” RBD. (TIF) [file ppat.1009352.s011.tif]

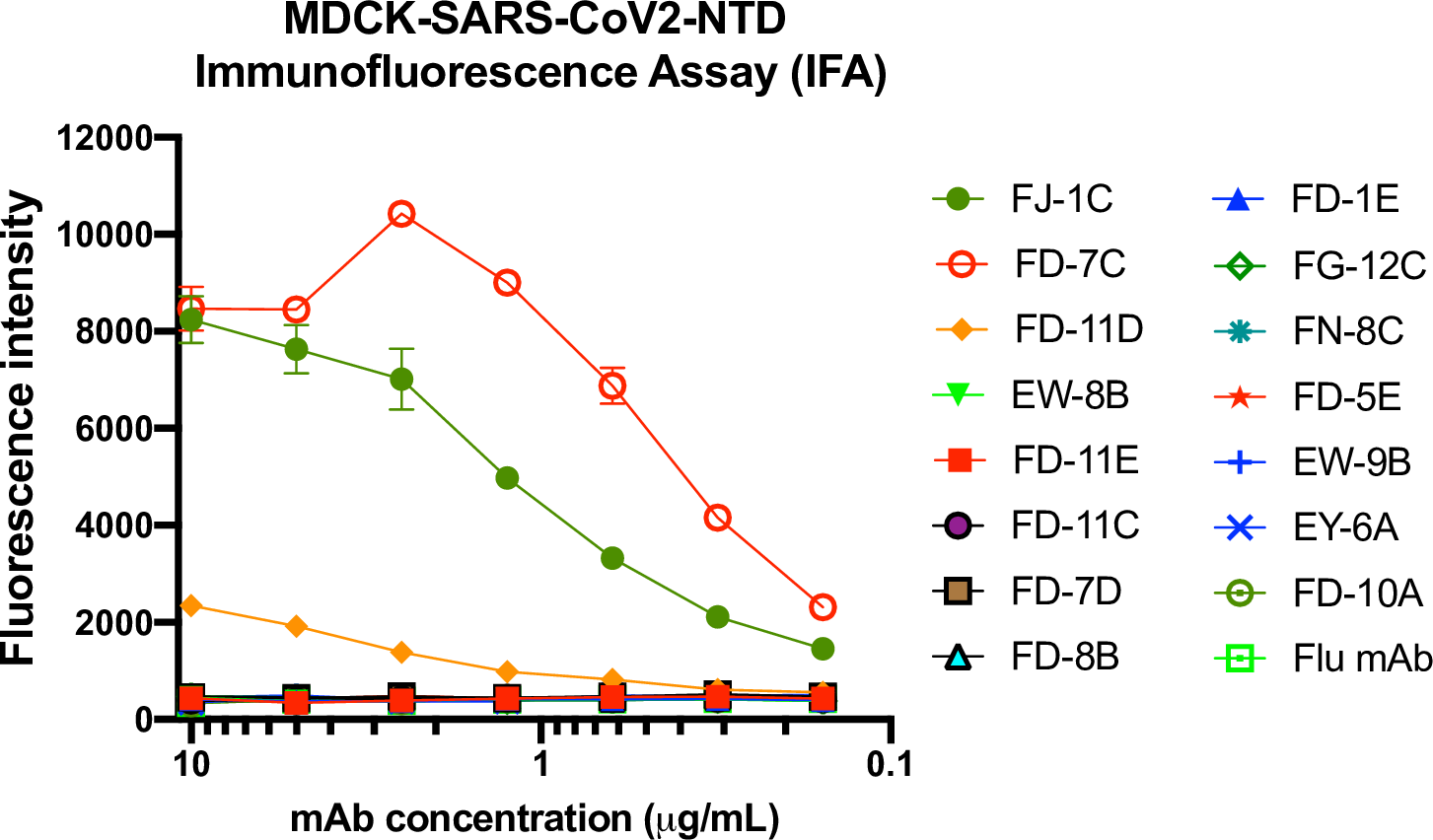

Supplement: S9 Fig — Anti-influenza neuraminidase Z3-B2 (Flu MAb) was included as control in the experiment. Each experiment was repeated twice. Values are presented as mean ± standard error of the mean. (TIF) [file ppat.1009352.s012.tif]

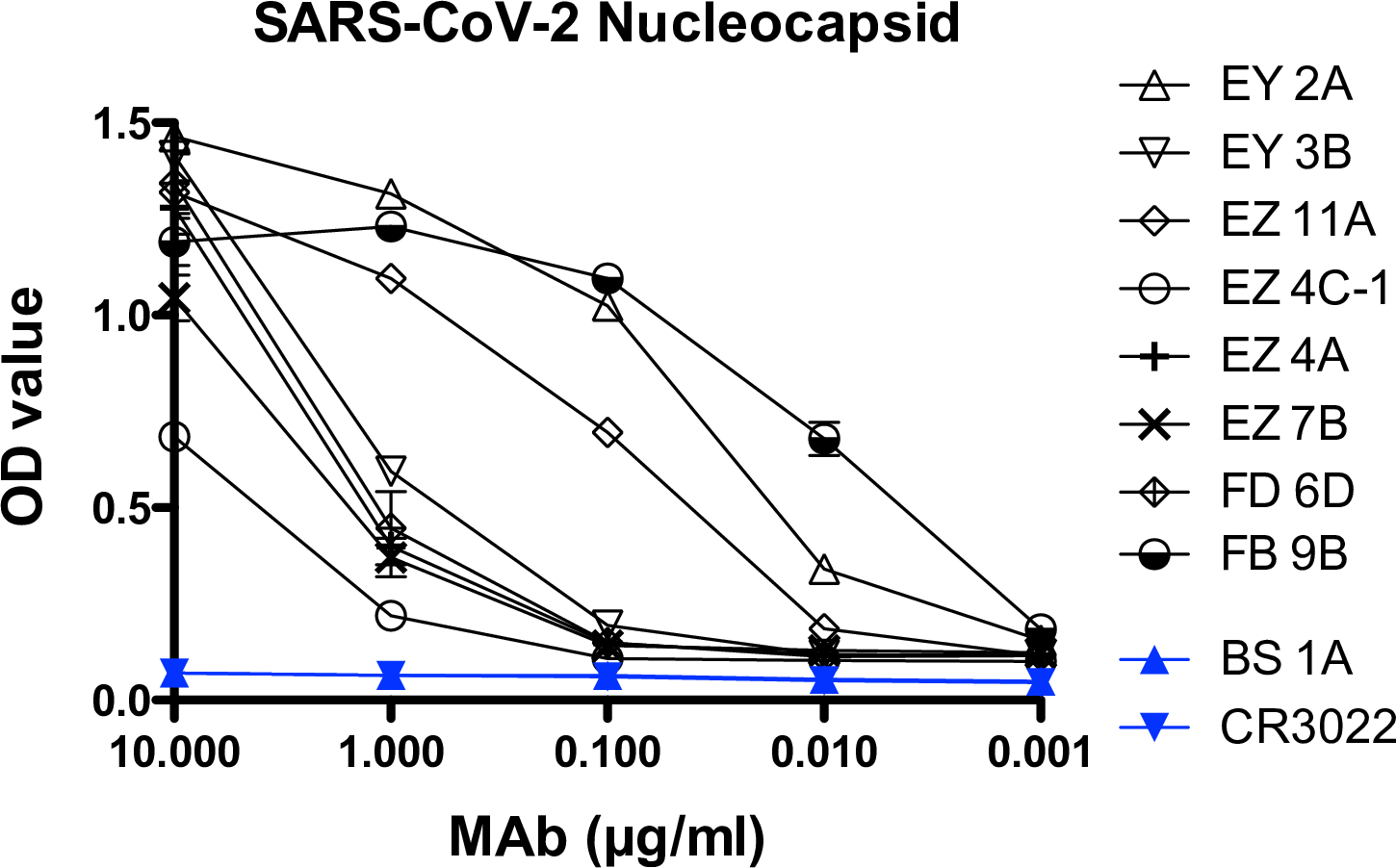

Supplement: S10 Fig — Anti-influenza H3 BS-1A and anti-SARS spike CR3022 MAbs were included as controls. Each experiment was repeated twice. OD450 values are presented as mean ± standard error of the mean. (TIF) [file ppat.1009352.s013.tif]

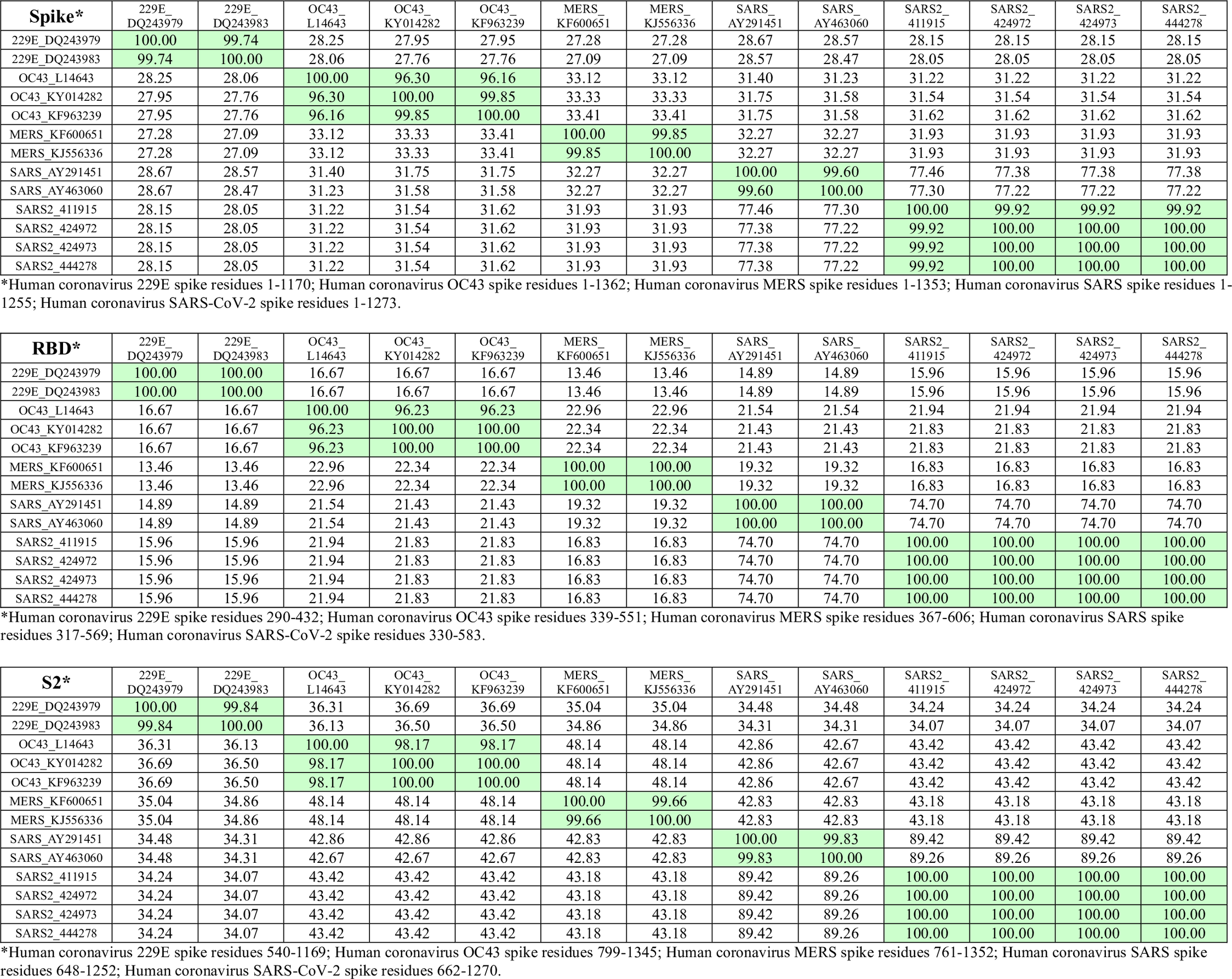

Supplement: S11 Fig — Sequences were retrieved from the Genbank database (DQ243979, DQ243983, KY014282, KF963239, L14643, KF600651, KJ556336, AY291451, AY463060) and the EpiFlu database of GISAID (EPI_ISL_411915, EPI_ISL_424972, EPI_ISL_424973, EPI_ISL_444278). (TIF) [file ppat.1009352.s014.tif]

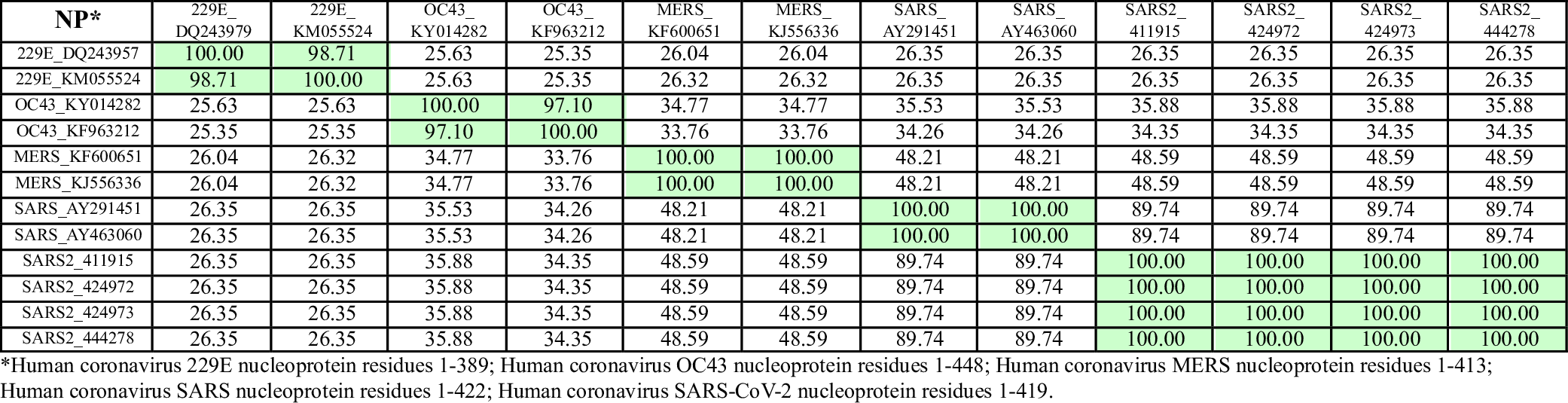

Supplement: S12 Fig — Sequences were retrieved from the Genbank database (DQ243957, KM055524, KY014282, KF963212, KF600651, KJ556336, AY291451, AY463060) and the EpiFlu database of GISAID (EPI_ISL_411915, EPI_ISL_424972, EPI_ISL_424973, EPI_ISL_444278). (TIF) [file ppat.1009352.s015.tif]
