# Supplementary figures and images for: Correction: Breadth and function of antibody response to acute SARS-CoV-2 infection in humans
Source: PLoS Pathog. 2021 Dec 10;17(12):e1010148. doi: 10.1371/journal.ppat.1010148 (PMC8664163; doi:10.1371/journal.ppat.1010148)

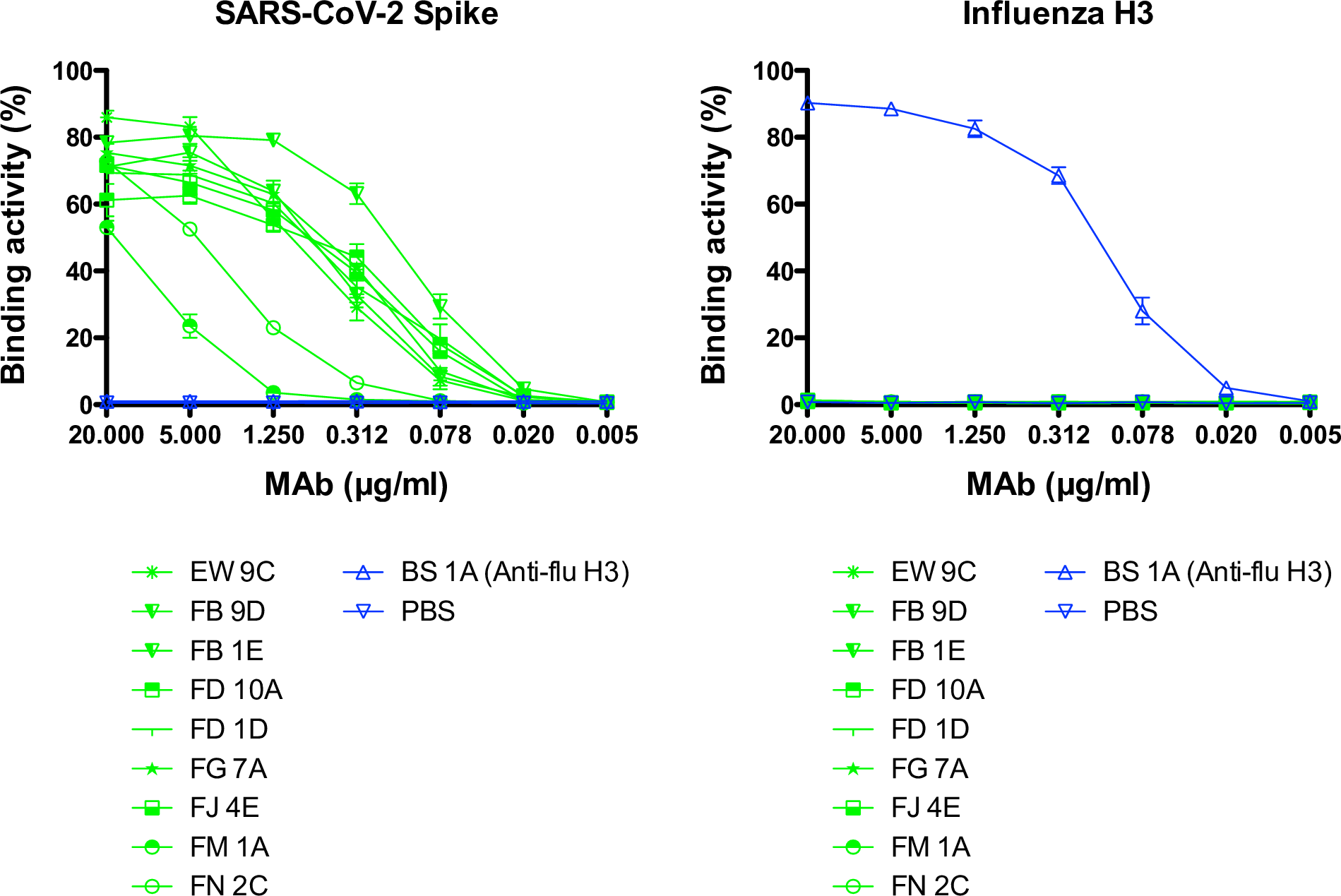

Supplement: S2 Fig — We produced MDCK-Spike by stably transducing parental MDCK-SIAT1 cells with cDNA expressing full-length unmutated SARS-CoV-2 spike glycoprotein. MDCK-H3 cells were stained in the control experiment. Anti-influenza H3 MAb BS-1A was included as an antibody control. Each experiment was repeated twice (n = 2). The binding percentage was presented as mean ± standard error of the mean. (TIF) [file ppat.1010148.s001.tif]
